# Demonstrating the Electronic Consent Process for Emergency Surgical Patients

**DOI:** 10.7759/cureus.72392

**Published:** 2024-10-25

**Authors:** David Fellows, Giles Bond-Smith

**Affiliations:** 1 General Surgery, Oxford University Hospitals NHS Foundation Trust, Oxford, GBR

**Keywords:** consent and capacity in uk law, electronic consent, general emergency surgery, quality improvement (qi), surgical informed consent

## Abstract

Introduction

Patient consent for surgery is a vital part of the surgical pathway from both a patient safety and medicolegal point of view. Improvement in documenting the full consent process in the electronic patient record (EPR), alongside the standard completion of the paper consent form in a surgical emergency unit (SEU), to comply with the Montgomery ruling of informed consent, has become of paramount importance. The aim of this project was to improve the documentation of the full consent process by creating an electronic template that was easy for the consenting clinician to use and provided a robust record of the bespoke consent process for the individual patient.

Methods

In July 2020, clinicians in the SEU were asked to begin documenting the patient's consent process in the EPR. The number of documented electronic consent processes (ECPs) of patients who underwent emergency general surgery during a two-week period was then captured between October and November 2020. The second cycle involved creating a single consent template, allowing the ECP to be easily documented in one template for all the common emergency surgery operations. All clinicians who were responsible for surgical consenting were asked to use the electronic template as part of the consent process. The rate of electronic consent was recorded over a second two-week period, six months later, in May 2021.

Results

From the first cycle, 78 patients were identified as undergoing emergency surgery, of which 13% (N=10) had an electronic entry regarding the consent process fully documented and personalised to the patient. From the second cycle, 64 patients were identified. The rate of documented ECPs increased to 77% (N=49).

Conclusion

This project has successfully improved the electronic demonstration of a consent process, bespoke to the individual, in accordance with the Montgomery ruling, by implementing an easy-to-use template on EPR. This is a significant step towards full digital consent in the future.

## Introduction

Consent for surgical procedures is a vital and medico-legally mandated step of the surgical process for patients. In the UK law, informed surgical consent is legally mandated. The process of informed surgical consent involves an appropriately trained clinician discussion with the patient and a resultant consent form signed by both the healthcare professional and the patient. The discussion that takes place includes the reasons for the proposed treatment, the intended benefits, and the possible risks. Historically, judgement on whether the clinician had discussed enough of the relevant risks to the patient was tested on the Bolam test when the clinician acted in line with a reasonable body of medical opinion [1]. Although the Bolam test used to be the legal framework for the consent process, guidance from regulatory bodies such as the General Medical Council (GMC) aimed to keep patients at the centre of the conversation, with their wishes and beliefs playing a key role in the consent process [2].

In 2015, a landmark case changed the UK law around consent for medical treatment. Mrs. Montgomery gave birth in 1999, and her baby suffered serious disabilities as a result of shoulder dystocia, a serious complication of vaginal delivery, during the birth. Mrs. Montgomery was diabetic, which increases the risk of shoulder dystocia; however, she was not informed of these risks during her pregnancy. She sought to seek compensation for her child's injuries. The Montgomery vs. Lanarkshire Health Board case ruled that the adequacy of consent would no longer be assessed using the Bolam test. Instead, it ruled that healthcare providers must provide the patient with information about all material risks, which are risks that a reasonable person would attach significance to the situation of the patient. This has switched the focus from what clinicians would deem important to what the patient would feel is important.

In the Montgomery court case, Paragraph 87 states, "The doctor is therefore under a duty to take reasonable care to ensure that the patient is aware of any material risks involved in any recommended treatment, and of any reasonable alternative or variant treatments. The test of materiality is whether, in the circumstances of each case, a reasonable person in the patient's position would be likely to attach significance to the risk, or the doctor is or should reasonably be aware that the particular patient would be likely to attach significance to it" [3].

This change in law has required a change in attitude towards the surgical consenting process. Patients are now more involved in the discussions regarding their treatment than ever before. The physical act of obtaining written consent has not changed, with physical surgical consent forms still common practice in most NHS trusts, some using electronic forms in the same format, stating the intended procedure, benefits, risks, and signature from the consenting clinician and patient. In elective settings, the conversation of the consenting process, including clinician and patient thought process and decision-making, is often apparent in outpatient clinic letters. However, this documentation in emergency surgical settings is usually lacking. Digital electronic patient records (EPRs) are becoming standard practice throughout NHS trusts, with consent forms also moving to electronic for many trusts. In this trust, paper consent forms were still in use during this project. One aim of this project was to begin the transition to electronic consenting by introducing a departmental policy to document the consent conversation on the patient's EPR. Improvement in documenting the full consent process in the EPR, alongside the standard completion of the paper consent form on a surgical emergency unit (SEU), to comply with the Montgomery ruling of informed consent, became of paramount importance.

This project aimed to ensure that all patients who underwent emergency general surgery in a major trauma centre had their full consent discussion documented in their patient records.

This project and the results were presented in poster format at the National QIP conference in July 2021.

## Materials and methods

This project was conducted in the SEU at the John Radcliffe Hospital, Oxford University Hospitals NHS Foundation Trust, Oxford, United Kingdom. In July 2020, clinicians in the SEU were asked to begin documenting the patient's electronic consent process (ECP) into the EPR. To aid this process, three templates were created for the more routine and common procedures, which were 'Diagnostic laparoscopy +/- appendicectomy +/- proceed', 'Examination under anaesthesia of rectum +/- incision and drainage of perianal abscess', and 'Incision and drainage of abscess' to aid this process. These procedures were chosen as they were thought to make up the majority of emergency surgical procedures on the SEU.

The templates included the same information as the paper consent form: the patient's name and age, the procedure's name, a statement that indications and alternatives have been discussed, and the risks. It also included extra details, including the patient's occupation, and a space for additional comments where clinicians were encouraged to document other aspects of the conversation or patient factors that were personalised to the patient, such as questions that the patient had or co-morbidities. The addition of the patient's occupation and space for additional comments made it unique to each patient and showed that patient factors such as co-morbidities were being taken into account, ensuring the ECP was documented in line with the Montgomery ruling.

Clinicians consenting to the SEU were asked to include the ECP for all surgical procedures, not just the templated ones. The rate of ECP being documented for patients undergoing emergency surgery was evaluated. Patients were identified by looking at the emergency surgical theatre list each day over a two-week period between October and November 2020.

The inclusion criteria encompassed all adult patients who consented to a general surgical emergency procedure on the SEU using consent form 1. All general surgical emergency procedures were included. Patients who consented to a general surgical procedure but then subsequently did not undergo surgery for any reason were included in the study, as the focus of this project was the consent process, not patient outcomes.

The exclusion criteria comprised patients who did not consent using consent form 1 (i.e., lacked the capacity to consent for themselves for any reason) and patients who underwent a surgical procedure under a different speciality other than general surgery (e.g., vascular).

The data were reviewed by calculating the percentage of patients with completed ECP in their records.

For the second cycle, to improve the rate of ECP documentation, a single consent template was created and uploaded to the trust EPR system, which included all of the previous consent templates as well as 'Diagnostic laparoscopy +/- cholecystectomy +/- proceed' and 'Diagnostic laparotomy +/- proceed'. These were chosen due to their representation of a significant proportion of surgical procedures from the obtained data. The template had a space for additional comments to document the specifics of the conversation. This allowed the ECP to be easily documented for all the common emergency surgery operations in one template, with options to remove non-applicable procedures and risks as appropriate.

All members of the surgical team working on the SEU were educated about the template and the departmental policy of documenting the ECP in the patient record. This was communicated to staff via email, and a visual guide to accessing and using the template on EPR was created and circulated via email. The rate of electronic consent over a second two-week period was recorded in May 2021, six months after the first cycle of data collection.

The data were again reviewed by calculating the percentage of patients who had a completed ECP in their records. Then, both cycles were analysed using the chi-square test of independence to test for statistical significance.

This project was registered locally as a Quality Improvement Project, number 6708.

## Results

From the first cycle, 92 patients were identified from the emergency surgical theatre list, of which 14 were excluded, leaving 78 patients who fit the inclusion criteria. Of these, 13% (N=10) of patients had an electronic entry in their record of the consent process fully documented and personalised to the patient. The breakdown of the types of procedures revealed that laparoscopic cholecystectomy was the most common procedure, followed closely by diagnostic laparoscopy. The three templates covered 45% of the captured patients (Table 1).

**Table 1:** Cycle 1 type and number of surgical procedures consented for and the percentage of those who had ECP documented ECP: electronic consent process

Procedure Consented For	Number of Patients	Number of ECP (%)
Laparoscopic cholecystectomy	25	3 (12%)
Diagnostic laparoscopy	22	6 (27%)
Incision & drainage of abscess	8	0 (0%)
Examination under anaesthesia of the rectum	5	0 (0%)
Laparotomy	7	0 (0%)
Laparoscopic colectomy	5	0 (0%)
Open hernia repair	2	0 (0%)
Colonic stent insertion	2	0 (0%)
Laparoscopic diverticular abscess drainage	1	1 (100%)
Laparoscopic deroofing of liver cyst	1	0 (0%)
Total	78	10 (13%)

From the second cycle, 83 patients were identified from the emergency surgical theatre list, of which 19 were excluded, leaving 64 patients. The fully documented, informed, and personalised consent rate increased to 77% (N=49). All of the ECP documentation was done using the new template provided. The breakdown of the procedure types found that laparoscopic cholecystectomy was again the most common procedure, followed closely by diagnostic laparoscopy. The template covered 92% of the captured procedures (Table 2).

**Table 2:** Cycle 2 type and number of surgical procedures consented for and the percentage of those who had ECP documented ECP: electronic consent process

Type of Procedure	Number of Patients (N)	Number of ECP (%)
Laparoscopic cholecystectomy	24	20 (83)
Diagnostic laparoscopy	20	17 (85)
Laparotomy	10	8 (80)
Examination under anaesthesia of the rectum	2	1 (50)
Incision & drainage of abscess	2	2 (100)
Open hernia repair	2	0 (0%)
Laparoscopic colectomy	2	1 (50)
Laparoscopic hiatus hernia repair	1	0 (0%)
Laparoscopic defunctioning loop ileostomy	1	0 (0%)
Total	64	49 (77%)

Statistical analysis using the chi-square test of independence found a statistically significant difference in terms of ECP documentation improvement between cycle 1 and cycle 2 (p < .05).

## Discussion

This project successfully achieved an increase in the rate of ECP documentation in a busy SEU. The results from the first cycle show that the templates were not capturing the most common surgical procedures; however, 40% of ECPs completed were for procedures that did not have a template, and there were none completed for incision and drainage (I&D) of abscess or examination under anaesthesia (EUA) rectum, which did have templates. This showed that although the template may help with the ECP documentation, it was not essential, and staff education and compliance were the likely limiting factors.

The second cycle showed a significant increase in ECP completion from 13% to 77%. The updated single template included surgical procedures, covering 92% of those captured during the two-week cycle, which contributed to this increase. Similar to the first cycle, one ECP was conducted for a procedure that was not listed on the template, showing that it was simple for the clinician to amend the template as needed for extra procedures.

The Montgomery case in 2015 shifted the focus of consenting patients towards information that the patient would reasonably attach significance to rather than information that medical professionals would agree is reasonable. Patients now have more autonomy and involvement than ever in the discussions regarding their treatment options and the risks associated with any proposed treatment.

Surgery of any kind can be an anxious time for a patient, especially emergency surgery when they are acutely unwell. Extensive research has examined how patient expectations and understanding can impact their outcomes [4]. Enabling patients to be better involved and educated on their treatment, with greater choice, will undoubtedly improve their experience of their surgical journey and, as a result, improve outcomes. Although the documentation of the consent process does not replace the physical consent form, which is signed by both the patient and the clinician, it does provide proof of the conversation that has taken place.

This project has successfully improved the electronic demonstration of a consent process, bespoke to the individual, in accordance with the Montgomery ruling. Digital EPRs have improved and streamlined patient documentation, and this project has been a significant step towards full digital consent in the future, compliant with the Montgomery ruling.

The limitations of this project were staff-specific. Due to the six-month period between the two data collection cycles, some clinical staff had left or started between cycles, mostly due to rotational training. This meant that the new staff, who started after the first cycle but before the second, were less aware of the project implementation and, therefore, less likely to ensure the ECP was documented. Another limitation was the range of surgical procedures included in the templates. The original three templates covered less than half of the captured surgical procedures. The updated template covered 92% of the procedures captured, which is a significant increase. However, the absence of the other 8% of procedures from the template may have contributed to a lack of ECP.

Due to the nature of working in emergency general surgery, clinicians are often under time pressures to complete tasks such as consent for surgery, and adding an extra step to the process posed a slight challenge to some staff. However, using a quick and easy-to-use template, resulting in a great increase between cycles and a rate of >75%, showed that this is a very attainable task.

Consideration can be made to expand the number of procedures on the template to ensure that ECP for a greater range of procedures is documented. Future work can look at how the individualised consent process taking place in a busy SEU impacts patient experiences and satisfaction outcomes of their overall surgical journey. Similar projects would greatly benefit other surgical specialties, especially in emergency settings.

## Conclusions

This project has successfully improved the electronic documentation of surgical consent for patients undergoing emergency general surgical procedures. Templates can be created and utilised by clinicians, making the process efficient, reducing workload and time spent, and overcoming potential engagement issues. Documenting individualised discussions with patients and showing patient engagement in the entire process shows compliance with the Montgomery ruling. This is a significant step towards full digital consent in the future, in accordance with the Montgomery ruling, improving medicolegal documentation for surgical patients.

## Appendices

Appendix 1: Cycle 1, Template 1

Procedure: Diagnostic Laparoscopy +/- Appendicectomy +/- Proceed

I have consented XXX, who is an XXX-year-old job description for a **Diagnostic Laparoscopy +/- Appendicectomy +/- Proceed.**

I have explained the procedure to the patient. I have discussed indications for surgical management and the availability of alternative treatments.

I discussed the following risk factors involved in the procedure, including but not limited to:

Generic:

·       Post-operative pain

·       Infection (superficial wound/intra-abdominal collections)

·       Bleeding

·       Damage to underlying structures (e.g. bowel, bladder, blood vessels, ureters)

·       Blood clot formation (DVT/PE)

·       Cardiorespiratory complications (CVA/MI/Pneumonia)

·       Port site hernia

Specific:

·       Stumpitis

·       Leak

·       Conversion to open

·       Bowel resection +/- stoma

Possible additional procedures:

·       Blood transfusion

·       Drain insertion

·       Catheter

Additional comments (if any):

Consented by: XXX

Appendix 2: Cycle 1, Template 2

Procedure: EUA Rectum + Incision and Drainage of Perianal Abscess

I have consented *XXX* who is a *XXX*-year-old *job description* for **EUA Rectum + Incision and Drainage of Perianal Abscess. **

I have explained the procedure to the patient. I have discussed indications for surgical management and the availability of alternative treatments.

I discussed the following risk factors involved in the procedure, including but not limited to:

Generic:

·       Post-operative pain

·       Infection (superficial wound)

·       Bleeding

·       Damage to underlying structures

·       Blood clot formation (DVT/PE)

·       Cardiorespiratory complications (CVA/MI/Pneumonia)

Specific:

·       Damage to anal sphincters/incontinence

·       Recurrence of abscess

·       Need for further procedures

·       Packing of abscess cavity

Possible additional procedures:

·       Drain insertion

·       Seton insertion

·       Fistulotomy

Additional comments (if any):

Consented by: *XXX*

Appendix 3: Cycle 1, Template 3

Procedure: Incision and Drainage of Abscess

I have consented *XXX* who is a *XXX*-year-old *job description* for **Incision and Drainage of XXX Abscess**. 

I have explained the procedure to the patient. I have discussed indications for surgical management and the availability of alternative treatments.

I discussed the following risk factors involved in the procedure, including but not limited to:

Generic:

·       Post-operative pain

·       Infection (superficial wound)

·       Bleeding

·       Damage to underlying structures (e.g. blood vessels, nerves)

·       Blood clot formation (DVT/PE)

·       Cardiorespiratory complications (CVA/MI/Pneumonia)

Specific:

·       Recurrence of abscess

·       Need for further procedures

·       Packing of abscess cavity

Additional comments (if any):

Consented by: *XXX*

Appendix 4: Cycle 2, Single Template

Procedure: (Insert procedure name here)

I have consented *XXX* who is a *XXX*-year-old *job description* for a (Insert procedure name here)

I have explained the procedure to the patient. I have discussed indications for surgical management and the availability of alternative treatments.

I discussed the following risk factors involved in the procedure, including but not limited to:

Generic:

·       Post-operative pain

·       Infection (superficial wound/intra-abdominal collections)

·       Bleeding

·       Damage to underlying structures (e.g. bowel, bladder, blood vessels, ureters)

·       Scar formation

·       Blood clot formation (DVT/PE)

·       Cardiorespiratory complications (CVA/MI//TIA/Pneumonia)

·       Port site hernia

·       Incisional hernia

·       Need for further procedures

(delete as appropriate):

Diagnostic laparoscopy +/- appendicectomy +/- proceed

Specific:

·       Stumpitis

·       Leak of bowel contents

·       Conversion to open

·       Bowel resection +/- stoma

Possible additional procedures:

·       Blood transfusion

·       Drain insertion

·       Catheter

EUA rectum +/- Incision and Drainage of Perianal Abscess

Specific:

·       Damage to anal sphincters/incontinence

·       Recurrence of abscess

·       Need for further procedures

·       Packing of abscess cavity

Possible additional procedures:

·       Blood transfusion

·       Drain insertion

·       Seton insertion

·       Fistulotomy

Incision and Drainage of XXX Abscess

Specific:

·       Recurrence of abscess

·       Need for further procedures

·       Packing of abscess cavity

Possible additional procedures:

·       Blood transfusion

·       Drain insertion

·       Catheter

Diagnostic laparoscopy +/- cholecystectomy +/- proceed

Specific:

·       Bile leak

·       Biliary tree damage

·       Conversion to open

Possible additional procedures:

·       Blood transfusion

·       Drain insertion

·       Catheter

Diagnostic laparotomy +/- proceed

Specific:

·       Need for further procedures

·       Bowel resection +/- stoma

·       Anastomotic leak

Possible additional procedures:

·       Blood transfusion

·       Drain insertion

·       Catheter

Additional comments (if any):

Consented by: *XXX*
